# Chirurgische Therapie des Ösophaguskarzinoms – Qualitätsindikatoren für Diagnostik und Therapie

**DOI:** 10.1007/s00104-020-01267-8

**Published:** 2020-09-02

**Authors:** Jens Hoeppner, Patrick Sven Plum, Heinz Buhr, Ines Gockel, Dietmar Lorenz, Michael Ghadimi, Christiane Bruns, Qualitätskommission der Deutschen Gesellschaft für Allgemein- und Viszeralchirurgie

**Affiliations:** 1 Klinik für Allgemein- und Viszeralchirurgie, Universitätsklinikum Freiburg, Medizinische Fakultät, Albert-Ludwigs-Universität Freiburg im Breisgau, Hugstetter Straße 55, 79106 Freiburg, Deutschland; 2 Klinik für Allgemein‑, Viszeral‑, Tumor- und Transplantationschirurgie, Universitätsklinikum Köln, Köln, Deutschland; 3 Deutsche Gesellschaft für Allgemein- und Viszeralchirurgie, Berlin, Deutschland; 4 Klinik für Viszeral‑, Thorax‑, Transplantations- und Gefäßchirurgie, Universitätsklinikum Leipzig, Leipzig, Deutschland; 5 Chirurgische Klinik I, Allgemein‑, Viszeral- und Thoraxchirurgie, Klinikum Darmstadt, Darmstadt, Deutschland; 6 Klinik für Allgemein‑, Viszeral- und Kinderchirurgie, Universitätsmedizin Göttingen, Göttingen, Deutschland

**Keywords:** Ösophaguskarzinom, Epidemiologie, Endoskopie, Mindestmenge, Prähabilitationsprogramme, Esophageal cancer, Epidemiology, Endoscopy, Minimum number, Prehabilitation program

## Abstract

**Hintergrund:**

Im Rahmen der Qualitätsinitiative der Deutschen Gesellschaft für Allgemein- und Viszeralchirurgie (DGAV) wurde eine Übersichtsarbeit auf der Grundlage einer systematischen Literatursuche verfasst und Empfehlungen für die aktuelle Diagnostik und Therapie des Ösophaguskarzinoms erarbeitet.

**Methoden:**

Die systematische Literatursuche erfolgte gemäß den PRISMA-Kriterien unter Verwendung der MEDLINE-Datenbank und wurde im März 2019 durchgeführt. Die Empfehlungen wurden auf der Grundlage von Abstimmungen in der DGAV formuliert.

**Ergebnisse und Schlussfolgerung:**

Operationen unterhalb der derzeit gültigen Mindestmenge sollten nicht mehr durchgeführt werden. Es spricht vieles dafür, die Mindestmenge in Deutschland auf ≥20 Resektionen/Jahr/Krankenhaus anzuheben, um die Qualität flächendeckend zu verbessern. Prähabilitationsprogramme mit Ausdauer‑, Kraft- und intensivem Atemtraining und Ernährungstherapie verbessern das Patientenoutcome. Die aktuelle Therapie des Ösophaguskarzinoms erfolgt stadienabhängig und umfasst die endoskopische Resektion bei (sub‑)mukosalen Low-risk-Tumoren (T1m1–3 bzw. T1sm1 „low risk“), die primäre Ösophagektomie bei submukosalen High-risk-Tumoren (T1a), Submukosakarzinomen (T1sm2–3) und T2N0-Tumoren, die multimodale Therapie mittels neoadjuvanter Radiochemotherapie bzw. perioperativer Chemotherapie und Operation bei fortgeschrittenen Befunden. Die Ösophagektomie wird gegenwärtig einzeitig als sog. Hybridverfahren (Laparoskopie und muskelschonende Thorakotomie) oder als total minimal-invasive Operation (Laparoskopie und Thorakoskopie) durchgeführt.

## Abstract (en)

**Background:**

Within the framework of the quality initiative of the German Society for General and Visceral Surgery (DGAV) a review article was compiled based on a systematic literature search. Recommendations for the current diagnostics and treatment of esophageal cancer were also elaborated.

**Methods:**

The systematic literature search was carried out in March 2019 according to the PRISMA criteria using the MEDLINE databank. The recommendations were formulated based on a consensus in the DGAV.

**Results and conclusion:**

Operations below the currently valid minimum quantity threshold should no longer be carried out. There are many indications that the minimum quantity in Germany should be raised to ≥20 resections/year/hospital in order to comprehensively improve the quality. Prehabilitation programs with endurance, strength and intensive breathing training as well as nutritional therapy improve patient outcome. The current treatment of esophageal cancer is stage-dependent and incorporates endoscopic resection of (sub)mucosal low-risk tumors (T1m1–3 or T1sm1 low risk), primary esophagectomy of submucosal high-risk tumors (T1a), submucosal cancer (T1sm2–3) and T2N0 tumors, multimodal treatment with neoadjuvant chemoradiotherapy or perioperative chemotherapy and operations for advanced stages. Esophagectomy is nowadays carried out in one stage as a so-called hybrid procedure (laparoscopy and muscle-preserving thoracotomy) or as a total minimally invasive operation (laparoscopy and thoracoscopy)

## Anmerkung der Autoren

Die Autoren möchten darauf hinweisen, dass dieser Artikel nicht als Leitlinie, sondern vielmehr als evidenzbasierter Expertenkonsens nach bester Evidenz und systematischer Literatursuche zu verstehen ist. Daher sei an dieser Stelle nochmals explizit auf die in diesem Zusammenhang existierende aktuelle S3-Leitlinie verwiesen [1].

## Hintergrund

Die chirurgische Resektion ist die zentrale kurative Therapiemodalität für nichtmetastasierte Ösophaguskarzinome. Insbesondere die Inzidenz des Adenokarzinoms des Ösophagus ist in den letzten zwei Dekaden ganz erheblich gestiegen und ist eine der am stärksten zunehmenden Krebserkrankungen in Europa und Nordamerika. Das klassische Plattenepithelkarzinom des Ösophagus macht ebenso einen weiterhin relevanten Teil der chirurgischen und kurativ behandelbaren Fälle aus. Durch diagnostische, chirurgisch technische und perioperative Verbesserungen konnten Morbidität und Mortalität der anspruchsvollen Ösophagusresektion in den letzten 20 Jahren deutlich verringert werden. Die onkologische Langzeitprognose der Erkrankung nach Resektion konnte durch Einführung neoadjuvant radiochemotherapeutischer und perioperativ chemotherapeutischer Behandlungsstrategien ebenfalls deutlich verbessert werden.

Die Autoren haben im Rahmen der Qualitätsinitiative der Deutschen Gesellschaft für Allgemein- und Viszeralchirurgie (DGAV) eine Übersichtsarbeit auf der Grundlage einer systematischen Literatursuche verfasst und Empfehlungen für die aktuelle Diagnostik und Therapie des Ösophaguskarzinoms erarbeitet.

## Material und Methoden

Die systematische Literatursuche erfolgte gemäß den PRISMA-Kriterien (Preferred Reporting Items for Systematic Reviews and Meta-Analyses) unter Verwendung der MEDLINE-Datenbank (https://www.ncbi.nlm.nih.gov/pubmed/) und wurde im März 2019 durchgeführt [2]. Die dabei zu betrachtenden Themenkomplexe („perioperative multimodale onkologische Therapiekonzepte“, „perioperatives Management“, „stadienabhängige kurative Therapie und Operationsindikation“ und „Tumordiagnostik bei Primärstaging und Restaging, Krankenhausstruktur“) wurden in jeweils eigenen Suchabfragen zusammengefasst und anschließend hieraus die relevanten Publikationen extrahiert. Um eine ausreichende Aktualität der Daten zu gewährleisten, wurde ein Suchzeitraum vom 01.01.2010 bis zum 13.03.2019 gewählt. Die gewählten Suchbegriffe entsprachen dabei den PRESS-Kriterien (Peer Review of Electronic Search Strategies).

Insgesamt wurden initial anhand der Kriterien 6879 Artikel identifiziert. Es konnten 5588 Duplikate nachgewiesen werden, sodass für die weiteren Analysen 1291 Artikel zur Verfügung standen. Nach Sichtung des Titels und/oder des Abstracts wurden letztlich 255 Artikel für die Volltextuntersuchung genutzt, von denen wiederum 25 Texte als Metaanalysen exkludiert wurden. Für die vorliegende Metaanalyse wurden final 230 Originalarbeiten herangezogen.

## Mindestmengen und Zentralisierung der Ösophaguschirurgie

Die in Deutschland geltende Mindestmenge von 10 komplexen Ösophagusresektionen/Jahr/Krankenhaus ist im europäischen Vergleich all der Länder, in denen Mindestmengen gelten, die niedrigste. Positive Auswirkungen einer Zentralisierung lassen sich an unterschiedlichen Parametern festmachen, wie die niederländische Qualitätsinitiative „Dutch Upper Gastrointestinal Cancer Audit“ (DUCA) eindrucksvoll belegte. Zwischen 2011 und 2014 wurde dieses webbasierte Qualitätsprogramm für die Therapie der Ösophagus- und Magenkarzinome eingeführt [3]. Für beide Tumorentitäten wurde die jährliche Mindestmenge auf 20 Resektionen/Jahr festgelegt. Im letzten Jahr der Einführung wurden knapp 100 % der operierten Patienten erfasst: Über den Beobachtungszeitraum sank die Krankenhausletalität von 8 % auf 4 % (Magenkarzinom) und blieb bei ca. 4 % stabil (Ösophaguskarzinom). Für das Ösophaguskarzinom hatten allerdings schon vorher Mindestmengen gegolten. In Dänemark wurde bei Eingriffen am Ösophagus die Anzahl der operierenden Institutionen 2006 von 26 auf nur 4 reduziert wurde. Die Krankenhausletalität wurde dadurch von 4,5 % auf 1,7 % gesenkt [4].

Schon für die in Deutschland geltende Mindestmenge von 10 Ösophagusresektionen/Jahr/Institution wurde in einer Analyse der Arbeitsgruppe um Mansky ein signifikanter Überlebensunterschied festgestellt. Danach lag die Krankenhausletalität in den Häusern <10/Jahr bei 12,1 % und für die >10/Jahr bei 9,2 % [5]. Anhand der deutschlandweiten Krankenhausabrechnungsdaten (DRG[„diagnosis related groups“]-Statistik) von 2010 bis 2015 wurde die Mengen-Ergebnis-Beziehung in Bezug auf die Krankenhaussterblichkeit, die Häufigkeit von Komplikationen und die Sterblichkeit von Patienten, bei denen Komplikationen auftraten, analysiert [6]. Insgesamt wurden 22.700 Behandlungsfälle mit komplexen Ösophaguseingriffen identifiziert. In Krankenhäusern mit sehr hoher Fallzahl (im Median 62 Behandlungsfälle pro Jahr) bestand im Vergleich zu Krankenhäusern mit sehr geringer Fallzahl (im Median 2 Behandlungsfälle pro Jahr) eine halb so große Wahrscheinlichkeit, infolge des Ösophaguseingriffs zu versterben (Odds Ratio [OR]: 0,50; 95 %-Konfidenzintervall [KI]: 0,42; 0,60). Die Krankenhaussterblichkeit der Patienten mit Komplikation lag in Krankenhäusern mit sehr hoher Fallzahl bei 12,3 %, in Krankenhäusern mit sehr geringer Fallzahl bei 20,0 %.

In der deutschen Statistik wurden alle komplexen Ösophagusresektionen erfasst – also auch Notfalleingriffe, z. B. wegen Perforation. Insofern sind die absoluten Zahlen nicht mit denen aus den Niederlanden oder Dänemark vergleichbar, wo nur elektive Resektionen dokumentiert wurden. Das Prinzip ist jedoch das gleiche: Komplikationen nach Ösophagusresektionen sind häufig [4] und die Möglichkeit, Patienten aus diesen kritischen Situationen zu retten, hängt von der Erfahrung („volume“) und der Ausstattung eines Zentrums ab. Ist beides nicht in ausreichendem Maß vorhanden, versterben die Patienten an den Folgen der Komplikationen signifikant häufiger („failure to rescue“).

## Tumordiagnostik

Die exakte Diagnostik des Ösophaguskarzinoms ist unabdingbar für die Therapieentscheidung. Neben der Anamnese und der körperlichen Untersuchung sind die Ösophagogastroduodenoskopie (ÖGD) mit Biopsie, der endoskopische Ultraschall (EUS) und die Computertomographie (CT) die drei wichtigsten Untersuchungstechniken in der Diagnostik.

### Ösophagogastroduodenoskopie mit Biopsie

Durch die hochauflösende ÖGD wird die direkte Tumorvisualisierung und Lokalisierung mit Höhen- und Größenangabe und die Entnahme von Biopsien ermöglicht. Es sollte weiterhin eine zirkuläre Lokalisationsangabe, der Stenosierungsgrad sowie die Höhe des oberen Ösophagussphinkters und der Z‑Linie bzw. des Zwerchfelldurchtritts dokumentiert werden. Die ÖGD mit Biopsie erreicht die höchste Sensibilität und Spezifität für einen Tumornachweis im oberen Gastrointestinaltrakt. Die Methode ist breit verfügbar und zeigt Komplikationsraten von 1 ‰ und eine Letalität <0,1 ‰ [7]. Biopsien sollten immer aus allen verdächtigen Läsionen entnommen und getrennt asserviert und beurteilt werden. Die Ausdehnung einer evtl. vorhandenen Barrett-Metaplasie sollte ebenfalls inklusive Biopsie erfasst werden. Sofern endoskopisch-makroskopisch der Verdacht auf einen malignen Prozess besteht, aber eine negative Histologie vorliegt, sollte eine Reendoskopie mit multiplen Rebiopsien erfolgen.

Zur Verbesserung der HD-Weißlicht-Endoskopie (HD-WLE) existieren Verfahren, die die Sensitivität verbessern sollen. Beschrieben sind die Chromoendoskopie, die computerbearbeitete virtuelle Chromoendoskopie („narrow-band-imaging“ u. a.) und die Vergrößerungsendoskopie (Endomikroskopie). Eine Metaanalyse, welche 11 randomisierte kontrollierte Studien (RCTs) einschlossen, untersuchte, ob die klassischen und die virtuellen Chromoendoskopieverfahren die Ergebnisse der HD-WLE bei der Aufdeckung von Ösophagusneoplasien beim Barrett-Ösophagus verbessen können. Dabei verbesserten die Chromoendoskopie und die virtuellen Verfahren die bioptisch überprüfte Aufdeckungsraten von HG-IEN (hochgradige intraepitheliale Neoplasie) und Frühkarzinomen um bis zu 34 % (KI 20–56 %, *p* < 0,0001; [8]). Ein Einsatz dieser Verfahren wird daher von der deutschen Leitlinie empfohlen.

Submukosale Läsionen stellen den Untersucher vor besondere Probleme bezüglich der Dignität der Läsion. Durch besondere Tiefenbiopsietechniken („deep biopsy“, „bite-on-bite biopsy“) kann die Erfolgsrate der Biopsie gesteigert werden bei gleichzeitig guter Blutungskontrolle und niedrigem Perforationsrisiko [9, 10].

### Computertomographie

Nach der histologischen Sicherung eines Karzinoms sollte sich eine CT von Thorax, Abdomen und ggf. des Halses (bei hochsitzenden Tumoren) mit oralem und intravenösem Kontrastmittel anschließen. Das CT ermöglicht eine relativ gute Aussage über eine Metastasierung in parenchymatösen Organen wie Leber und Lunge, über die Position des Tumors, über eine mögliche Organüberschreitung und einen Einbruch in Gefäße oder Nachbarorgane. Eine Aussage bezüglich einer Lymphknotenmetastasierung ist nur eingeschränkt möglich, da die Größe von Lymphknoten nicht immer mit der Dignität korreliert [1].

### Endoskopischer Ultraschall

Beim Nachweis von Fernmetastasen kann auf eine EUS-Untersuchung verzichtet werden. Sofern jedoch keine Fernmetastasen vorliegen, sollte zum Staging eine EUS-Untersuchung durchgeführt werden. Der EUS hat den Vorteil einer sehr hohen lokalen Ortsauflösung und bietet daher die höchste Treffsicherheit aller Verfahren zur Beurteilung der lokalen Infiltrationstiefe (T-Kategorie) und zur Beurteilung von Metastasen in regionäre Lymphknoten [11, 12]. Das Verfahren erreicht bei der Beurteilung der T‑Kategorie (für höhere T‑Stadien) eine Sensitivität von 92 % und eine Spezifität von 99 %. In Kombination mit einer Feinnadelbiospie können auch regionäre Lymphknoten abgeklärt werden und die Sensitivität und Spezifität des Verfahrens in der Beurteilung der Lymphknoten auf bis zu 97 % bzw. 96 % gesteigert werden [11, 12].

### PET-CT

Zum Einsatz einer Positronenemissionstomographie(PET)-CT beim Primärstaging existieren mehrere Studien, die zeigen konnten, dass bei ca. 16–32 % der Patienten weitere Lymphknoten- oder Fernmetastasen diagnostiziert werden können, die durch EUS und CT nicht gefunden wurden [13–16]. Die Sensitivität und Spezifität der PET-CT wird dabei von einigen Autoren mit bis zu 91 % bzw. 94 % angegeben [15].

### UICC-TNM-System

Entsprechend der deutschen Leitlinie sollte der histopathologische Befund der Operationsresektate die Größe, den histologischen Typ und die Art der neoplastischen Läsion mit Lokalisation in Bezug zum gastroösophagealen Übergang beschreiben. Es sollte eine Einteilung ins aktuelle UICC(Union for International Cancer Control)-TNM-System erfolgen. Wichtig sind hierbei Grading, Lymphgefäß- und Veneninfiltration, Resektionsränder oral, aboral und zirkumferenziell sowie der Lymphknotenstatus. Sofern eine neoadjuvante Therapie erfolgte soll eine Regressionsgrading erfolgen.

### Response auf die neoadjuvante Therapie

Derzeit existiert keine Untersuchung, die die Ansprechrate auf die neoadjuvante Therapie ausreichend valide abschätzen kann [17]. Daher ist aktuell nach neoadjuvanter Therapie die Operation mit anschließender histopathologischer Aufarbeitung des Resektates weiterhin der Goldstandard einer kurativen Therapie. Im Rahmen des Restagings sollten Kontraindikationen für die Operation und das Auftreten von Fernmetastasen ausgeschlossen werden. Hierzu erscheinen eine erneute ÖGD und ein CT von Thorax und Abdomen ausreichend.

## Stadienabhängige kurative Therapie und Operationsindikation

Die kurative Therapie des Ösophaguskarzinom variiert in Abhängigkeit vom jeweiligen Tumorstadium: Bei Frühkarzinomen (pT1) ist eine lokale Therapie mittels endoskopischer Mukosaresektion (EMR) bzw. Submukosadissektion (ESD) vertretbar, sofern keine Kontraindikationen wie Submukosainfiltration >500 µm, lymphovaskuläre Infiltration (L1, V1) oder G3/4-Differenzierung vorliegen [1]. Nichtsdestotrotz ist ein nodales Metastasierungsrisiko von bis zu 34 % bei zunehmender Infiltrationstiefe von pT1-Befunden mit Beteiligung der tieferen Schichten der Submukosa (sm2–3) zu beachten (Tab. 1; [18, 19]). Daher ist in solchen Fällen die Ösophagektomie mit regionärer Lymphadenektomie zu empfehlen. In einer retrospektiven Untersuchung zeigten sich keine Unterschiede im Überleben zwischen solchen Patienten mit primärer Operation bei Frühkarzinom im Vergleich zur operativen Resektion nach endoskopischer Intervention. Jedoch war die Rezidivrate nach alleiniger endoskopischer Resektion signifikant höher [20].

**Table 1:** 

Parameter	Literatur	Evidenzlevel	Kategorie
Mindestfallzahl von ≥20 Resektionen/Jahr/Krankenhaus	[3–6]	3	Struktur
Einsatz von Chromoendoskopie zum Nachweis intraepithelialer Neoplasien	[8]	1b	Prozess
Einsatz von Tiefenbiopsietechniken wie „deep biopsy“, „bite-on-bite biopsy“	[9, 10]	2a	
Kombination von EUS (ggf. mit Feinnadelbiopsie) zum Nachweis lokoregionärer Lymphknotenmetastasen	[11, 12]	1b	
Einsatz des PET-CT zum Nachweis von Lymphknoten- bzw. Fernmetastasen	[13–16]	2a	
Präoperatives Scoring des Patienten hinsichtlich – Kardiovaskulärer, pulmonaler, hepatischer und metabolischer Funktion – Compliance (GA) – Ernährungszustand (BMI, NRS, MNA)	[1, 54, 57, 59–61, 66]	2b	
Einsatz von Prähabilitation und ERAS zur Verbesserung der postoperativen Rekonvaleszenz	[64, 65, 67, 68]	1b	
EMR bzw. ESD bei – Submukosainfiltration <500 µm – Fehlender lymphovaskulärer Infiltration (L1, V1) – Differenzierung <G3 → Low-risk-Tumoren (T1m1–2, T1sm1 „low risk“)	[1, 20, 69–73]	2a	
Primäre Ösophagektomie bei → (Sub)mukosalen High-risk-Tumoren (T1a, T1sm1) → Submukosatumoren (T1sm2–3) → T2N0-Tumoren	[74]	3	
Multimodale Therapiekonzepte bei T2N^+^- bzw. T3N0/N^+^-Tumoren – Neoadjuvante Radiochemotherapie – Perioperative Chemotherapie → und operative Resektion	[49, 51, 75, 76]	1b	
Minimal-invasive Operation zur Komplikationsverringerung – Hybridverfahren – Total minimal-invasive Verfahren	[77, 78]	1b	
– Definitive Radiochemotherapie bei irresektablen Tumoren bzw. funktionell-inoperablen Patienten (mit der Möglichkeit der Salvage-Resektion)	[52, 53, 79]	2a	
Optimierung des intraoperativen Airway-Managements durch – Senkung des Tidalvolumens – PEEP-Optimierung – Rektrutierungsmanöver – Restriktion der Volumentherapie Peridualanalgesie	[62, 80]	3	
Strukturierte Komplikationserfassung gemäß Esophagectomy Complications Consensus Group	[27, 28]	2b	
Senkung von – Mortalität – Anastomoseninsuffizienz/postoperativer Komplikationen durch Behandlung in spezialisierten Zentren	[3–5, 26, 29]	3	Ergebnis

*BMI *Body-Mass-Index, *ESD *endoskopische Submukosadissektion, *EMR *endoskopische Mukosaresektion, *ERAS *„enhanced recovery after surgery“, *EUS *endoskopischer Ultraschall,* GA *geriatrisches Assessment,* MNA* Mini Nutritional Assessment,* NRS *Nutritional Risk Score, *PET-CT *Positronenemissionstomographie-Computertomographie,* PEEP *„positive end-expiratory pressure“

Analog hierzu sollten T2-Ösophagustumoren ohne Hinweis auf lokoregionäre Metastasierung direkt der Operation zugeführt werden oder aber alternativ wie bei T2-Tumoren mit lokalem Lymphknotenbefall bzw. T3- und T4a-Tumoren die multimodale Therapie mit perioperativer Chemotherapie bzw. neoadjuvanter Strahlenchemotherapie indiziert werden (Tab. 1; [21–24]).

Die operative Resektion eines Ösophaguskarzinoms stellt einen der komplexesten viszeralchirurgischen Eingriffe dar und sollte dementsprechend an einem Zentrum mit entsprechender Expertise erfolgen, da hierdurch die postoperative Mortalität deutlich gesenkt werden kann [25]. Durch entsprechende Spezialisierung liegen die Raten der 30-Tage- und 90-Tage-Mortalität bei 2,4 % bzw. 4,5 % [26].

Zudem ist hier die standardisierte Erfassung möglicher Komplikationen gemäß der sog. Esophagectomy Complications Consensus Group (ECCG) gewährleistet [27]. Dabei werden typische intra-/postoperative Komplikationen wie Anastomoseninsuffizienzen, Interponatnekrosen, Lymphfisteln, Affektionen des N. laryneus recurrens, Vorhoffflimmern oder Pneumonie systematisch erfasst und gemäß der Clavien-Dindo-Klassifikation kategorisiert [27, 28]. Anastomoseninsuffizienzen werden auch in Zentren im Durchschnitt bei 11,4 % der Patienten beobachtet [26].

### Rekonstruktionstechniken

Im Laufe der Zeit haben sich verschiedene Rekonstruktionstechniken etabliert: In Abhängigkeit von der Lage der Anastomose differenziert man zwischen hoch-thorakaler (Ivor Lewis; 56,3 %) und zervikaler (McKeown‑)Ösophagektomie (43,7 %; [26, 29]). Wurde vormals der Großteil der Eingriffe offen-chirurgisch durchgeführt, wird heutzutage nahezu die Hälfte der Eingriffe in minimal-invasiver Operationstechnik (MIE) vorgenommen [26]. Auch gibt es mittlerweile Kombinationsverfahren wie die Hybridösophagektomie, bei der die abdominelle Gastrolyse und Lymphadenektomie laparoskopisch erfolgt und die thorakale Resektion/Rekonstruktion offen-chirurgisch [30–33].

Die Datenlage hinsichtlich der Überlegenheit eines der genannten Verfahren ist uneinheitlich: Beide Rekonstruktionen (thorakal vs. zervikal) scheinen die gleiche klinische Sicherheit für den Patienten zu bieten, jedoch ergaben Metaanalysen, dass die Ivor-Lewis-Ösophagektomie hinsichtlich des Krankenhausaufenthaltes und Blutverlustes möglicherweise überlegen ist [34, 35]. Systematische, prospektive Daten stehen noch aus. Bezüglich des Einsatzes minimal-invasiver Techniken konnte für die Hybridösophagektomie eine signifikante Überlegenheit in den Ergebnissen in der postoperativen Morbidität bei gleichwertigen onkologischen Ergebnissen prospektiv-randomisiert bewiesen werden [28]. Die robotisch-assistierte Ösophagektomie stellt die jüngste Weiterentwicklung hinsichtlich der minimal-invasiven Operationsmethodik dar [36–39].

### Lymphadenektomie

Im Rahmen der Ösophagektomie erfolgt die 2‑Feld-Lymphadenektomie des Abdomens und unteren Mediastinums mit der Resektion von mindestens 20 bis 30 Lymphknoten. Mittlerweile ist hier jedoch davon auszugehen, dass nicht die Gesamtzahl der resezierten Lymphknoten von prognostischer Relevanz ist, sondern vielmehr das Verhältnis tumorpositiver zu negativen Lymphknoten („lymph node ratio“; [40]).

#### Infobox 1 Operative Therapie

- Die Ösophagektomie wird heutzutage einzeitig als sog. Hybridverfahren (Laparoskopie und muskelschonende Thorakotomie) oder als total minimal-invasive Operation (Laparoskopie und Thorakoskopie) durchgeführt.
- Rekonstruktives Grundprinzip ist die Wiederherstellung der Passage im oberen Gastrointestinaltrakt mit Bildung eines Schlauchmagens und Ösophagogastrostomie.
- Seltener erfolgen Dünn und Dickdarminterpositionen zur Passagerekonstruktion (Ösophagojejunostomie bzw. Ösophagokolostomie).
- In Abhängigkeit von der Lage der Anastomose unterscheidet man zwischen der Ösophagektomie nach Ivor-Lewis (intrathorakale Anastomose) und der Resektion nach McKoewn (zervikale Anastomose).
- Im Rahmen der Ösophagektomie erfolgt die 2‑Feld-Lymphadenektomie des Abdomens und unteren Mediastinums mit der Resektion von mindestens 20 bis 30 Lymphknoten.
- Bei gastroösophagealen Übergangstumoren vom Typ AEG II ist alternativ die Resektion mittels transhiatal erweiterter Gastrektomie und Rekonstruktion mittels Ösophagojejunostomie eine Option.

### Tumoren des ösophagogastralen Übergangs

Eine besondere Herausforderung stellt die Resektion der Tumoren des ösophagogastralen Übergangs (Adenokarzinome des ösophagogastralen Übergangs, AEG) dar. AEG-II-Tumoren können neben der Ösophagektomie auch mittels transhiatal erweiterter Gastrektomie (TEG) versorgt werden. Hinweise aus Beobachtungsstudien, ob der rein abdominelle Zugang der TEG zu weniger pulmonalen bzw. funktionellen Komplikationen führt und/oder mit einer höheren Lebensqualität bei vergleichbaren onkologischen Ergebnissen verbunden ist, werden aktuell in der internationalen CARDIA-Studie prospektiv randomisiert überprüft werden [41–43].

In Infobox 1 sind die operativen Strategien beim Ösophaguskarzinom zusammengefasst.

## Perioperative multimodale onkologische Therapiekonzepte

Die perioperative Chemotherapie und die neoadjuvante Radiochemotherapie verbessern das Langzeitüberleben beim nichtmetastasierten Ösophaguskarzinom in den Stadien T2–T4 NX M0 im Vergleich zur alleinigen chirurgischen Resektion signifikant ([44–46]; Tab. 2). Die Operation wird in den multimodalen Therapieschemata 3 bis 8 Wochen nach Ende der neoadjuvanten Therapie durchgeführt und beeinflusst die postoperative Morbidität und Mortalität im Vergleich zur alleinigen Operation nicht [44–49]. Die postoperative Mortalität in den aktuellen multimodalen Protokollen beträgt 4–8 % [44–46, 49]. Für ein verlängertes Intervall zwischen dem Ende der neoadjuvanten Behandlung und dem Zeitpunkt der Operation gibt es aktuell keine überzeugende Evidenz [50]. Beim Plattenepithelkarzinom der Speiseröhre wird durch den aktuellen internationalen Standard mit einer Radiochemotherapie (41,4 Gy plus Carboplatin/Paclitaxel) das mediane Gesamtüberleben auf 82 Monate gegenüber 21 Monaten (Hazard Ratio [HR] 0,48) bei der alleinigen chirurgischen Therapie gesteigert [51]. Beim Adenokarzinom der Speiseröhre verbessert die neoadjuvante Radiochemotherapie das mediane Gesamtüberleben auf 43 Monate gegenüber 27 Monaten (HR 0,73; [51]). Im Gegensatz zum Plattenepithelkarzinom wird beim Adenokarzinom das Langzeitüberleben auch durch die perioperative Chemotherapie verbessert. Im Vergleich zur alleinigen Operation zeigen die prospektiv-randomisierten Studien MAGIC (Epirubricin, Cisplatin, 5‑FU) und FFCD9703 (Cisplatin, 5‑FU) signifikante Überlebensvorteile mit 5‑Jahres-Überlebensraten von 36 vs. 23 % (HR 0,75) bzw. 38 vs. 24 % (HR 0,69; [44, 45]). Der aktuelle chemotherapeutische Standard beim Adenokarzinom wird durch die perioperative FLOT-Therapie (5-Fluoruracil, Leukovorin, Oxaliplatin, Docetaxel) mit einem medianen Gesamtüberleben von 50 Monaten gebildet [49]. Welchem der beiden genannten multimodalen Therapiekonzepte im Hinblick auf Kurz- und Langzeitergebnisse beim Adenokarzinom der Vorzug zu geben ist, ist ungeklärt und international umstritten. Die Frage ist Gegenstand der in Deutschland aktuell laufenden multizentrischen ESOPEC-Studie [21].

**Table 2:** 

Tumorstadium	TNM-Stadium	Behandlung
Mukosakarzinome	cT1a cN^–^ „low risk“	Endoskopische Resektion
Submukosakarzinome	cT1b sm1 cN^–^ „low risk“	
Mukosakarzinome	cT1a cN^–^ „high risk“	Thorakoabdominelle Ösophagusresektion + 2-Feld-Lymphadenektomie
Submukosakarzinome	cT1b sm2/3 cN^–^	
Lokalisierte Karzinome	cT1 cN^+^	
	cT2 cN^–^	
Lokal fortgeschrittene Karzinome	cT1‑2 cN^+^	Thorakoabdominelle Ösophagusresektion + 2-Feld-Lymphadenektomie *plus AC/PEC*: neoadjuvante Radiochemotherapie (z. B. 41,4 Gy, Carboplatin + Paclitaxel) oder *AC*: perioperative Chemotherapie 3‑Fach-Kombination (Docetaxel, Platin + Flouropyrimidin)
	cT3 cN^–/+^	
	cT4a cN^–/+^	

*AC/PEC* Adenokarzinom/Plattenepithelkarzinom

Bei intendiert definitiv radiochemotherapeutisch behandelten Ösophaguskarzinomen mit persistierendem Tumor oder lokalem Tumorrezidiv kann eine Salvage-Resektion und damit ebenfalls ein multimodales Therapiekonzept indiziert sein. Die zu diesem Konzept vorliegenden Daten zeigen eine höhere Morbidität und Mortalität (9,5 vs. 4,5 %) bei der Salvage-Resektion im Vergleich zur geplanten postneoadjuvanten Resektion [52]. Die vorliegenden Daten zum Langzeitoutcome des Salvage-Operationskonzepts sind unzureichend, weisen aber auf zu mindestens gleichwertige Überlebenszeiten im Vergleich zur geplanten Operation hin [53].

## Perioperatives Management

### Risikoevaluation und -stratifizierung

Eine differenzierte Risikoevaluation und -stratifizierung, möglichst vor Beginn einer neoadjuvanten Therapie, ist essenziell für ein erfolgreiches Outcome nach onkologischer Ösophagektomie. Hierzu haben sich, neben einer umfassenden Abklärung der kardiovaskulären, pulmonalen, hepatischen und metabolischen Funktion [1], validierte Scoringsysteme etabliert mit Korrelation zwischen präoperativem Status und postoperativer Morbidität und Letalität. Dabei ist O‑POSSUM derzeit der am besten validierte, international angewandte klinische Risikoscore, der zur Prädiktion der postoperativen Morbidität und Mortalität nach Ösophagektomie zur Verfügung steht [54].

Neben dem Risikoscoring ist die Einschätzung der Compliance des Patienten für den postoperativen Verlauf entscheidend. Seine aktive Mitarbeit ist essenziell, um insbesondere die pulmonale Morbidität durch intensives Atemtraining zu vermindern.

Ältere Patienten haben bekanntermaßen ein größeres Risiko für postoperative Komplikationen nach Ösophagektomie [55]. Hier wird zusätzlich ein prätherapeutisches geriatrisches Assessment (GA) empfohlen, um ggf. eine risikoadaptierte Therapiemodifikation (z. B. der neoadjuvanten Therapie) durchzuführen [56] – basierend auf der individuellen „frailty“ (Gebrechlichkeit) bzw. „prefrailty“ sowie der Funktions- und Fähigkeitseinschränkungen im GA (gemäß den Empfehlungen der AG „Geriatrische Onkologie“ der Deutschen Gesellschaft für Hämatologie und Medizinische Onkologie [DGHO]).

Pragmatisch und zeiteffizient durchzuführen sind präoperativ der 6‑minute-walk-Test (6 MWT; [57]) sowie der Shuttle-walk-Test (SWT; [58]). Ein weiteres einfaches und genaues Testverfahren zur exakten Beurteilung der Leistungsfähigkeit stellt die Spiroergometrie dar. Hochrisikopatienten sollten mittels Ergometrie identifiziert werden [59]. Die präoperative Leistungsfähigkeit stellt einen wichtigen Prädiktor der postoperativen Prognose bei Patienten mit Ösophaguskarzinom dar [58, 60].

### Ernährungsstatus

Ein prätherapeutisches Screening beim Ösophaguskarzinom muss hinsichtlich Malnutrition und Katabolie basierend auf dem vorhandenen Ausmaß der Dysphagie erfolgen [1]. Die Erfassung des BMI (Body-Mass-Index), des NRS (Nutritional Risk Score), des MNA (Mini Nutritional Assessment) und einer potenziellen Sarkopenie impliziert die Applikation oraler Trinklösungen, supportiver enteraler Nahrungssubstanzen bzw. evt. einer i.v. Supplementierung.

Risikopatienten mit einem Gewichtsverlust von >10 % in den vorausgegangenen 3 Monaten, einem BMI <18,5 kg/m^2^ oder einer Serumalbuminkonzentration <30 g/l sollten präoperativ mithilfe einer Ernährungstherapie vorbereitet werden [61].

### Optimiertes anästhesiologisches Management

Die Beatmung mit niedrigem Tidalvolumen, Optimierung des PEEP („positive end-expiratory pressure“) sowie die routinemäßige Anwendung von Rekrutierungsmanövern reduzieren postoperative pulmonale Komplikationen. Die Flüssigkeitstherapie sollte eine Überwässerung vermeiden, welche ebenso einen bekannten Risikofaktor für pulmonale Komplikationen sowie Anastomoseninsuffizienzen darstellt. Die thorakale Periduralanalgesie reduziert die systemische inflammatorische Reaktion, vermindert pulmonale Komplikationen und verbessert die postoperative Schmerzkontrolle [62].

### Prähabilitation und „enhanced recovery after surgery“

Die funktionelle Rekonvaleszenz ist entscheidend für das postoperative Ergebnis. Dabei bedingt in der Ösophaguschirurgie die Prähabilitation zur Optimierung der funktionellen Reserven vor der Operation, z. B. im Rahmen der neoadjuvanten Therapie, eine beschleunigte Erholungsphase und eine Reduktion von Komplikationen. Zu den multimodalen Präkonditionierungskonzepten zählen eine Kombination aus Ausdauer‑, Kraft- und intensivem Atemtraining sowie einer Ernährungstherapie unter Berücksichtigung des individuellen präoperativen Leistungsniveaus und des Ernährungsstatus, idealerweise unter professionellem Monitoring.

„Enhanced-recovery-after-surgery“(ERAS)-Programme fokussieren gezielt die Steuerung einer standardisierten Behandlung nach der Operation mit dem Ziel der Minimierung postoperativer Komplikationen und der Beschleunigung der Rekonvaleszenz [63]. Während für den positiven Effekt der Prähabilitation vor der Ösophaguschirurgie evidenzbasierte Daten in der Literatur vorliegen [64, 65], sind die ERAS-Pathways zwar vielversprechend, um einen weiteren Benefit zu erzielen, wenngleich zum aktuellen Zeitpunkt große, prospektive Multicenterstudien mit entsprechender Evidenz fehlen.

## Fazit für die Praxis

- Es spricht vieles dafür, die Mindestmenge in Deutschland auf ≥20 Resektionen/Jahr/Krankenhaus anzuheben, um die Qualität (Senkung der Morbidität bzw. Mortalität) flächendeckend zu verbessern.
- Für die Diagnostik des Ösophaguskarzinoms sollte neben der Anamnese und der körperlichen Untersuchung die Durchführung einer Ösophagogastroduodenoskopie (ÖGD) mit Biposie, die endoskopische Sonographie (EUS) und die Computertomographie (CT) des Thorax/Abdomens durchgeführt werden.
- Die Therapie des Ösophaguskarzinoms erfolgt stadienabhängig und umfasst die endoskopische Resektion bei (sub-)mukosalen Low-risk-Tumoren (T1m1–3 bzw. T1sm1 low risk), die primäre Ösophagektomie bei submukosalen High-risk-Tumoren (T1a), Submukosakarzinomen (T1sm2–3) und T2N0-Tumoren, die multimodale Therapie mittels neoadjuvanter Radiochemotherapie bzw. perioperativer Chemotherapie und Operation bei fortgeschrittenen Befunden.
- Die intra-/postoperative Komplikationen wie Anastomoseninsuffizienzen, Interponatnekrosen, Lymphfisteln, Affektionen des N. laryneus recurrens, Vorhoffflimmern oder Pneumonie sollten gemäß der sog. Esophagectomy Complications Consensus Group (ECCG) standardisiert erfasst werden.
- Zur präoperativen Risikoabschätzung gehört die Untersuchung der kardiovaskulären, pulmonalen, hepatischen und metabolischen Funktion. Zudem sollte der Ernährungszustand des Patienten mittels BMI (Body Mass Index), NRS (Nutritional Risk Score) oder MNA (Mini Nutritional Assessment) erfasst und eine Malnutrition ggf. bereits präoperativ behandelt werden.
- Prähabilitationsprogramme mit Ausdauer‑, Kraft- und intensivem Atemtraining sowie einer Ernährungstherapie verbessern das Patientenoutcome deutlich.
- Intraoperativ profitiert der Patient von einer restriktiven Volumentherapie, niedrigem Tidalvolumen, Optimierung des PEEP („positive end-expiratory pressure“) sowie des Einsatzes der Periduralanalgesie.
